# The association of gender with receptive and distributive needle sharing among individuals who inject drugs

**DOI:** 10.1186/s12954-022-00689-3

**Published:** 2022-09-30

**Authors:** Katherine M. Rich, Julia Zubiago, Meghan Murphy, Rubeen Guardado, Alysse G. Wurcel

**Affiliations:** 1 Harvard Medical School, Boston, MA USA; 2 Division of Geographic Medicine and Infectious Diseases, Department of Medicine, Tufts Medical Center, Boston, MA USA; 3 Tufts University School of Medicine, Boston, MA USA

**Keywords:** Injection drug use, Harm reduction, HIV prevention, Women

## Abstract

**Background:**

Injection drug use and needle sharing remains a public health concern due to the associated risk of HIV, HCV and skin and soft tissue infections. Studies have shown gendered differences in the risk environment of injection drug use, but data are currently limited to smaller urban cohorts.

**Methods:**

To assess the relationship between gender and needle sharing, we analyzed publicly available data from the 2010–2019 National Survey on Drug Use and Health (NSDUH) datasets. Chi-square tests were conducted for descriptive analyses and multivariable logistic regression models were built adjusting for survey year, age, HIV status, and needle source.

**Results:**

Among the entire sample, 19.8% reported receptive needle sharing, 18.8% reported distributive sharing of their last needle, and 37.0% reported reuse of their own needle during last injection. In comparison with men, women had 34% increased odds (OR 1.34, 95% CI 1.11–1.55) of receptive needle sharing and 67% increased odds (OR 1.67, 95% CI 1.41–1.98) of distributive needle sharing. Reuse of one's own needle did not differ by gender.

**Conclusions:**

In this nationally representative sample, we found that women are more likely in comparison with men to share needles both through receptive and distributive means. Expansion of interventions, including syringe service programs, to increase access to sterile injection equipment is of great importance.

## Background

Rates of infections and related hospitalizations are rising among people who inject drugs (PWID) in large part driven by the opioid epidemic [1, 2] and the sharing of non-sterile injecting equipment. In particular, sharing of equipment increases the risk for HIV, hepatitis C (HCV), and skin and soft tissue infections (SSTIs) [3–5]. An analysis of county-level vulnerability identified over 200 counties across 26 states at high risk for an outbreak of HIV or HCV due to shared injection equipment [6]. The rapid outbreaks of HIV among networks of PWID in Indiana and Massachusetts further underscore this risk and the need for expansion of targeted harm reduction programs [5, 7].

Prior work has identified the impact of gender on needle and works sharing [8, 9]. Women are more likely to initiate injection drug use in the context of a relationship [10, 11], share needles the first time they ever inject [12, 13], and receive “assisted injection,” a practice that involves one individual injecting another [14, 15], The sexual and power dynamics of injection partner dyads and networks also has been shown to increase the risk environment of women who inject drugs [16]. Prior work by our research team found an increased rate of skin and soft tissue infections among women who engaged in sex work, but not men [17].

However, research on sharing injection equipment, to date, has been limited to smaller urban cohorts and precedes the current era of ubiquitous synthetics drugs. With the rise of fentanyl and other synthetic opioids alongside the shifting geo-demographic patterns of injection drug use, the gendered patterns of works sharing may have shifted. An updated and national assessment of the influence of gender on works sharing is warranted to help guide harm reduction programs and advocacy efforts. In this paper, we investigate if there is an association between gender and needle sharing in a national dataset from 2010 to 2019.

## Materials and methods

We analyzed ten years of data from the 2010–2019 National Survey on Drug Use and Health (NSDUH) publicly available datasets, which included responses from a total of 564,177 participants across those ten years. Only participants who reported any injection drug use (IDU) were included in our analysis sample.

Our primary outcome of interest was the report (yes/no) that the last needle used had been previously used by another individual (“receptive needle sharing”). Our secondary outcome of interest was report (yes/no) of giving the last needle they had used to someone else following injection (“distributive needle sharing”). The primary indicator of interest was gender, recorded by NSDUH interviewers as women or men. Since the creation of the original NSDUH survey, there has increased attention to the importance of asking about gender and sex in ways that are respectful and inclusive. Recent analyses of NSDUH reported on gender [18], as such we framed our analysis of the survey item as gender. Other indicators of interest in our analysis included age, race, ethnicity, self-reported HIV status, source of last needle, and survey year. Descriptive analyses with chi-square tests were conducted to examine differences by gender. Multivariable logistic regression models were then built to further examine the relationship between gender and (1) distributive needle sharing and (2) receptive needle sharing. Multivariable models were adjusted for year, race/ethnicity, age, HIV status, and needle source. These variables were chosen a priori guided by prior studies [8, 9, 19, 20]. The NSDUH uses a stratified cluster design to select a representative sample of non-institutionalized people living in the USA. To account for the complex cluster design, all analyses were weighted using the survey package in R (v.4.0.0) and the variance estimation variables and final analysis weights provided in each NSDUH dataset.

## Results

There were 7678 survey respondents who reported IDU (1.4% of NSDUH 2010–2019 responses). Most of the sample (79.5%) identified as non-Hispanic White, 42.2% were 50 years of age or older and most (98.5%) were HIV negative (Table 1). Half of participants reported obtaining the needle for their most recent IDU from a pharmacy (51.3%), and 5.4% reported obtaining the needle from a syringe service program (SSP). Nearly one in five respondents (19.8%) reported receptive needle sharing, and 18.7% reported distributive needle sharing. Two thirds (66%) of those who received a needle from another individual reported distributing the same needle following injection. About a third of all survey participants (37.0%) reported reuse of their own needle during last injection.

**Table 1:** Cohort characteristics stratified by gender (*n* = 7678)

	Male (*N* = 4571)	Female (*N* = 3107)	Overall (*N* = 7678)	*p* value
	Raw number (weighted %)			
*Age*				
12–17yo	131 (0.6%)	147 (1.2%)	278 (0.8%)	< 0.001
18–25yo	1106 (8.2%)	880 (11.6%)	1986 (9.3%)	
26–34yo	1001 (18.5%)	807 (23.0%)	1808 (19.9%)	
35–49yo	1301 (27.2%)	797 (29.1%)	2098 (27.8%)	
50 or older	1020 (45.6%)	463 (35.1%)	1483 (42.2%)	
*Race/ethnicity*				
Black (non-Hispanic)	251 (7.7%)	87 (3.4%)	338 (6.3%)	< 0.001
Hispanic	459 (9.8%)	289 (9.2%)	748 (9.6%)	
Other (non-Hispanic)	340 (4.2%)	316 (5.3%)	656 (4.6%)	
White (non-Hispanic)	3509 (78.3%)	2402 (82.1%)	5911 (79.5%)	
*HIV status*				
HIV−	4380 (98.2%)	3036 (99.1%)	7416 (98.5%)	0.051
HIV+	67 (1.8%)	17 (0.9%)	84 (1.5%)	
*Receptive sharing: last needle used by someone else prior to participant*				
Yes	678 (18.6%)	645 (22.2%)	1323 (19.8%)	0.026
No	3852 (80.9%)	2432 (77.2%)	6284 (79.7%)	
Don't know/refused	28 (0.5%)	16 (0.6%)	44 (0.5%)	
*Distributive sharing: last needle used by someone after participant*				
Yes	668 (16.4%)	702 (23.6%)	1370 (18.7%)	< 0.001
No	3836 (82.2%)	2352 (74.5%)	6188 (79.8%)	
Don't know/refused	54 (1.2%)	39 (1.9%)	93 (1.5%)	
*Needle reuse: participant reused their own needle*				
Yes	1598 (36.6%)	1257 (37.8%)	2855 (37.0%)	0.776
No	2920 (62.5%)	1815 (61.2%)	4735 (62.1%)	
Don't know/refused	40 (0.9%)	21 (0.9%)	61 (0.9%)	
*Needle source*				
Pharmacy	2383 (49.3%)	1540 (49.8%)	3923 (51.3%)	< 0.001
SSP	285 (4.9%)	215 (6.3%)	500 (5.4%)	
Bought on street	453 (10.4%)	252 (6.7%)	705 (9.2%)	
Friend/relative	696 (16.9%)	606 (22.8%)	1302 (18.8%)	
Got some other way	703 (17.9%)	455 (18.3%)	1158 (18.0%)	

Women were more likely to both report receptive (*p* = 0.026) and distributive needle sharing (*p* < 0.001) in bivariate analyses. Reuse of one’s own needle did not differ by gender. In multivariable modeling, women were significantly more likely to report receptive and distributive needle sharing, adjusted for age, needle source, HIV status, race and ethnicity, and year of survey (Table 2). In comparison with men, women had 34% increased odds (OR 1.34, 95% CI 1.12–1.59) of receptive needle sharing and 67% increased odds (OR 1.67, 95% CI 1.41–1.98) of distributive needle sharing.

**Table 2:** Multivariable models: factors associated with report of receptive needle sharing

	Receptive needle sharing aOR (95%CI)	Distributive needle sharing aOR (95%CI)
*Sex*		
Male	Ref	Ref
Female	**1.34 (1.12**–**1.59)**	**1.67 (1.41**–**1.98)**
*Age*		
12–17yo	**0.43 (0.26**–**0.71)**	0.60 (0.36–1.01)
18–25yo	**0.65 (0.50**–**0.86)**	**0.78 (0.62**–**0.97)**
26–34yo	**0.54 (0.43**–**0.68)**	**0.64 (0.50**–**0.82)**
35–49yo	**0.62 (0.49–0.80)**	**0.72 (0.57–0.92)**
50 or older	Ref	Ref
*Needle source*		
SSP	Ref	Ref
Pharmacy	**0.56 (0.36**–**0.86)**	**0.64 (0.43**–**0.96)**
Bought on street	**1.92 (1.17**–**3.15)**	**1.76 (1.09**–**2.85)**
Friend/relative	**1.66 (1.05**–**2.63)**	**1.66 (1.07**–**2.56)**
Got some other way	1.18 (0.75–1.85)	1.28 (0.82–1.99)
Year	**0.94 (0.91**–**0.98)**	**0.94 (0.91**–**0.97)**
*Race*		
Black (non-Hispanic)	0.95 (0.61–1.48)	0.99 (0.62–1.59)
Hispanic	1.26 (0.95–1.69)	1.31 (0.95–1.79)
Other (non-Hispanic)	0.80 (0.53–1.20)	1.02 (0.69–1.52)
White (non-Hispanic)	Ref	Ref
HIV positive	1.60 (0.81–3.17)	0.69 (0.32–1.50)

Multivariable logistic regression model included race/ethnicity (White non-Hispanic, Black Non-Hispanic, Other), HIV status, sex, age, needle source, and year of survey. Year was included as a continuous variable

## Discussion

In this nationally representative sample of PWID, we found that women were significantly more likely to report sharing of needles than men, both through receptive and distributive patterns. This finding may provide an explanation for the higher incidence of SSTIs that have been reported among women who inject drugs [17]. While the gender and characteristics of the needle sharing partner(s) are not reported in NSDUH, our results are suggestive that women who inject drugs may be at higher risk of bloodborne disease as a result of needle sharing practices.

Previous research has emphasized the role that social and environmental factors play in shaping injection behaviors for women. For instance, gendered violence and complex power dynamics has been shown to influence needle sharing practices, such that women are more likely to “go second on the needle” due to differential sexual, physical and environmental influences [16, 21, 22]. While gendered violence very likely contributes to our findings, our data also suggest a nuanced picture of injection practices among women. Women may be more likely to inject socially and provide needles to others. A majority of those in our sample who received a needle subsequently distributed or returned a needle, indicating individuals are injecting in pairs and/or larger networks. Social support and injecting with others present are protective factors against physical violence as well as fatal overdose, particularly with the rise of more potent synthetic drugs. Accordingly, interventions for risk reduction should balance the protective effects of injecting in groups with strategies to reduce needle sharing.

A secondary finding of our work is the substantial need for expanded harm reduction programs nationally, given the reported low frequency of obtaining a needle from an SSP (5.4%). Evidence-based harm reduction interventions are central to reducing needle sharing, decreasing transmission of HIV and HCV, and preventing unintentional overdose. Not only are SSPs able to provide sterile equipment, but these programs offer non-judgmental, free, and accessible testing and care services to PWID who may not contact other medical services. Secondary needle exchange programs from SSPs that provide individuals with sterile injection equipment for both the individual and their peer network may be a particularly effective strategy given our findings of sharing of injection equipment. Efforts to address barriers to accessing harm reduction programs will be critical to ensure that there are sites which are safe and accessible for all who need sterile injection equipment, including the expansion of safe consumption sites (or ‘overdose prevention sites’) [23–26]. Furthermore, an increased emphasis on addressing the specific needs of women who inject drugs will be crucial. Previous work has suggested the importance of integrated mental health services with harm reduction, as well as interventions that provided empowerment training to address gender-based violence [16, 27].

Our analysis is limited in that we can only assess behavior surrounding a participant’s last needle used which provides a limited snapshot into injection practices. Characteristics (e.g., gender, age, relationship with survey participant) of who the needle was shared with are not known. Additionally, given the NSDUH survey design, we were unable to assess sharing behaviors of nonbinary people, who may have distinct patterns of equipment sharing due to differing power dynamics and exposure to violence [28, 29]. Future research and expanded survey designs are crucial to better understand injection practices of all individuals. Another limitation of this dataset is that the NSDUH does not include those who are incarcerated and relies on people with an address or who are living in a shelter, which may not capture those who are street homeless and using substances. While the NSDUH has been shown to better represent PWID than other national surveys, our sample may have underestimated the prevalence of PWID given selection bias, as well as the stigma of reporting IDU behaviors [30]. This being said, in our analysis, we estimated the prevalence of PWID to be 1.4% of all surveyed participants in line with the most recent estimates of national PWID prevalence of 1.46% reported by Bradley and colleagues [31].

## Conclusions

Harm reduction strategies aimed at reducing high-risk injections and IDU-related infection prevention programs should focus efforts to include gender-specific components that address the needs of women who inject drugs. These findings are particularly important in the context of the continued opioid epidemic and rising use of synthetic drugs, including fentanyl.

## Data Availability

The datasets analyzed for the current study are publicly available and published by the National Survey on Drug Use and Health.

## Abbreviations

CI: Confidence interval
HCV: Hepatitis C virus
HIV: Human immunodeficiency virus
IDU: Injection drug use
OR: Odds ratio
PWID: People who inject drugs
NSDUH: National Survey on Drug Use and Health
SSP: Syringe service program
SSTIs: Skin and soft tissue infections
